# STEAP4 drives immune dysregulation in myocardial infarction–NSCLC comorbidity

**DOI:** 10.3389/fcell.2026.1862249

**Published:** 2026-06-30

**Authors:** Zhonghe Liu, Yuxi Yi, Wenwen Li, Zhexin Wang, Yukuan Lang, Lin Huang, Haoran Zhai, Liang Xu, Liang Chen, Xiaoke Chen, Shijie Fu, Zhonghao Tao

**Affiliations:** 1 Shanghai Lung Cancer Center, Shanghai Key Laboratory of Thoracic Tumor Biotherapy, Shanghai Chest Hospital, Shanghai Jiao Tong University School of Medicine, Shanghai, China; 2 Department of Radiation Oncology, Shanghai Chest Hospital, Shanghai Jiao Tong University School of Medicine, Shanghai, China; 3 Department of Respiration, Shanghai Chest Hospital, Shanghai Jiao Tong University School of Medicine, Shanghai, China; 4 Department of Thoracic Surgery, Shanghai Chest Hospital, Shanghai Jiao Tong University School of Medicine, Shanghai, China

**Keywords:** bioinformatics analyses, comorbidity phenomenon, experimental validation, immune microenvironment, STEAP4

## Abstract

**Introduction:**

Myocardial infarction (MI) and non-small cell lung cancer (NSCLC) are leading causes of morbidity and mortality worldwide, with increasing evidence pointing to a complex comorbidity between them. However, the immunological mechanisms linking these two diseases remain largely unexplored.

**Methods:**

We integrated bioinformatics analysis with in vivo experimental validation. MI datasets (GSE48060, GSE83500) and an NSCLC dataset (GSE19188) were obtained from the GEO database. A multilayer screening framework combining differential expression analysis, WGCNA, and machine learning algorithms (SVM-RFE, LASSO, and random forest) was applied to identify shared hub genes. A mouse model of MI and NSCLC comorbidity was established, and STEAP4 was functionally evaluated through gene knockout and siRNA mediated knockdown, with assessment of cardiac function, tumor burden, immune cell infiltration, and inflammatory cytokine levels.

**Results:**

Four hub genes, STEAP4, HP, TREM1, and CCR2, were identified, and their combined signature achieved excellent diagnostic performance (AUC = 0.987). In the comorbidity mouse model, STEAP4 was significantly upregulated in both cardiac and pulmonary tissues, correlating with elevated serum IL-6 and TNF-α levels. Immune profiling revealed increased neutrophil infiltration and reduced CD8^+^ T cells in the local microenvironment. Notably, STEAP4 knockout or knockdown significantly improved cardiac function, reduced myocardial fibrosis, suppressed tumor growth, and partially restored immune homeostasis.

**Discussion:**

These findings establish STEAP4 as a pivotal mediator of MI and NSCLC comorbidity, linking systemic inflammation with immune microenvironment dysregulation. Our multi-omics and functional validation approach provides a mechanistic framework for understanding cardiovascular-oncological comorbidity and positions STEAP4 as a potential diagnostic biomarker and therapeutic target for further investigation.

## Graphical Abstract

**Figure:**
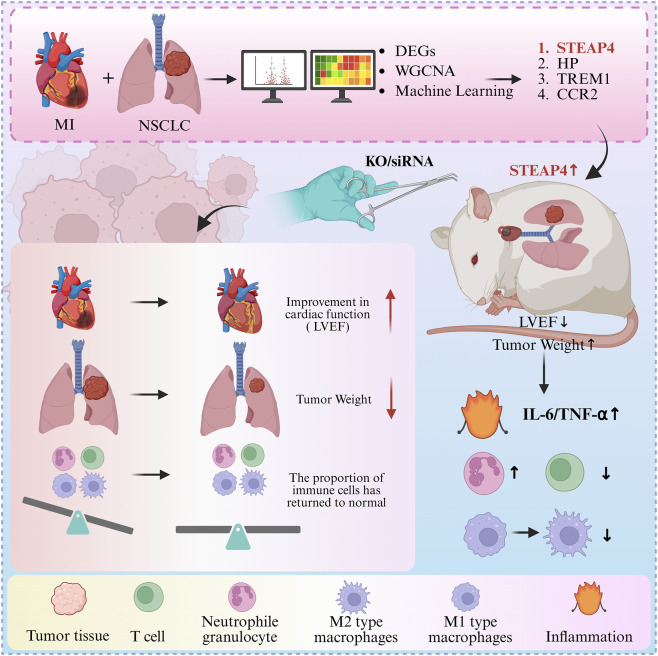
Schematic illustration of the mechanistic role of STEAP4 in the comorbidity of MI and NSCLC. The graphical abstract was created using BioRender and is published under an authorized license.

## 1 Introduction

Myocardial infarction (MI) is a potentially fatal ischemic heart disease resulting from the prolonged interruption of coronary blood flow, leading to necrosis of the heart. It is characterized by a sudden onset, often with a poor outcome, thus contributing to the global burden of morbidity and mortality. Epidemiological studies have indicated that MI remains a major contributor to disability and mortality worldwide (Saito et al., 2024; Chen et al., 2025). Despite advances in the care of cardiovascular diseases, MI still affects populations across the world at high rates (Salari et al., 2023; Upadhyaya et al., 2024). Although considerable advances have been made in reperfusion techniques and pharmacotherapy, the late consequences of MI, including heart failure and arrhythmias, remain major prognostic and quality of life problems (Kosugi et al., 2025; Wang et al., 2025). Significantly, there is increasing evidence to suggest that MI has effects beyond a local insult on the heart, and through a systemic inflammatory response and neuroendocrine dysfunction, it can have far-reaching effects on other body systems.

Epidemiological data indicate that NSCLC accounts for approximately 85% of lung cancer cases. Due to the high incidence and contribution to the burden of cancer, NSCLC has been identified as a major public health burden worldwide (Sun et al., 2021; Chen et al., 2026; Bo et al., 2024). Despite the improvements in the diagnostic and therapeutic measures, this cancer continues to afflict patients at high rates, resulting in a significant number of cancer-related deaths worldwide (Li et al., 2023; Kocarnik et al., 2022; Yu et al., 2026). The prognosis for patients with advanced-stage NSCLC remains poor. Though the treatment modalities for NSCLC, including surgical, radiation, targeted, and immunotherapy, have improved considerably, the survival benefits for patients with late-stage NSCLC remain limited. These poor clinical outcomes highlight the need for novel biomarkers and therapeutic strategies (Herbst et al., 2018; Skribek et al., 2022). Of particular importance is the fact that treatment-related adverse effects are a significant feature in NSCLC, and cardiovascular events are a major clinical concern in this context.

Recent clinical and epidemiological evidence has increasingly pointed to a complex comorbid relationship between MI and NSCLC. Emerging evidence indicates that these two conditions do not only share common risk factors but also interact at the molecular and immunological levels (Nandi et al., 2025; El-Rayes et al., 2025). On the one hand, patients with MI might be at a high risk of developing NSCLC, which could be partly attributed to the shared risk factors, including smoking, aging, and systemic inflammation. In addition, the systemic inflammation that characterizes the microenvironment following a myocardial infarction could be conducive for the development of cancer. Pro-inflammatory cytokines, such as IL-6 and TNF-α, produced during MI, play an essential role in the development of atherosclerosis and tumor cell proliferation and metastasis by activating oncogenic signaling pathways, including NF-κB and STAT3 (Reuter et al., 2010; Damiano et al., 2025). On the other hand, patients with NSCLC, particularly those undergoing chemotherapy, radiotherapy, or targeted therapy, present a greater incidence of cardiovascular complications, including MI, which is considered one of the most severe cardiovascular complications. The cardiotoxicity of some antineoplastic agents also contributes to this severity (Hardy et al., 2010; Herbach et al., 2022). Certain chemotherapeutic agents have the potential to induce myocardial damage directly or indirectly, thus making the patient more susceptible to ischemic complications (Ma Y. et al., 2025). Furthermore, cytokines and exosomes produced by the tumor can affect vascular and myocardial function, thus creating a bidirectional pathophysiological interplay between cardiovascular and pulmonary function (Ma C. et al., 2025; Mei et al., 2025).

Significantly, the link between MI and NSCLC is not limited to the causative-effect relationship but is part of an intricate network of interrelated pathophysiological mechanisms, including chronic inflammation, immune system dysregulation, metabolic reprogramming, and oxidative stress. In this respect, the dysregulated activation of immune cells, cytokine interactions, and epigenetic regulation of metabolic pathways represent the molecular basis of the cross-talk between MI and NSCLC (Nandi et al., 2025; Zheng et al., 2024). Despite the recognition of the clinical co-existence of MI and NSCLC, the underlying molecular interaction network for this comorbidity has yet to be elucidated. The goal of the current research is to identify the key biomarkers and pathways, which is essential for further understanding and designing novel early diagnostic tools and comprehensive treatment options for patients with co-existing MI and cancer.

Our aim was to integrate bioinformatics analysis with experimental validation to identify the common molecular mechanisms of MI and NSCLC. To do so, we employed a multi-omics data mining approach, along with sophisticated machine learning techniques, to identify genes that are simultaneously involved in the pathogenesis of MI and NSCLC. This strategy resulted in the identification of STEAP4 (Six-Transmembrane Epithelial Antigen of Prostate 4) as a key candidate gene. Further *in vivo* experiments were conducted to provide evidence of the functional role of STEAP4 in the promotion of MI-NSCLC comorbidity. In addition to differential expression profiling, the study further explored the regulatory role of STEAP4 on immune cell infiltration, cytokine network remodeling, and disease-associated metabolic reprogramming. The overall effect of this integrative approach is an improved current understanding of the molecular interplay associated with the co-existence of MI-NSCLC, while the findings can create a theoretical basis for the development of more precise diagnostic markers and therapeutic strategies, which can be used for the stratification of patients with complex cardiovascular-oncological comorbid conditions.

## 2 Methods

### 2.1 Data collection and flow chart

**FIGURE 1:**
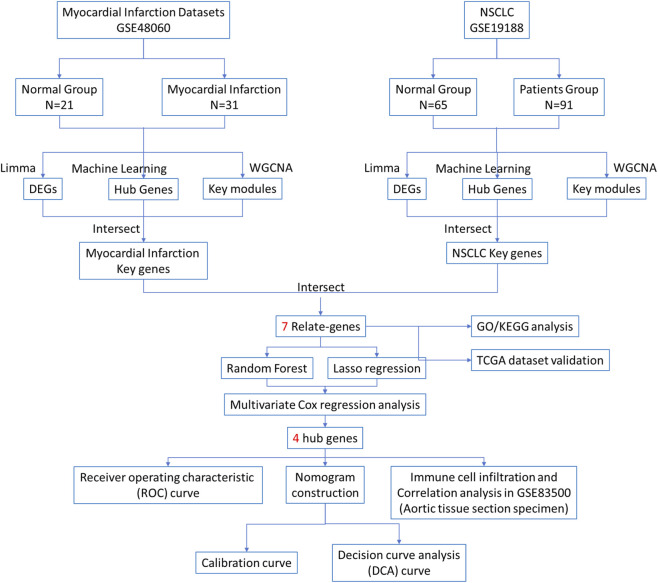
Flowchart of our research.

Gene expression datasets were obtained from the GEO database, a widely used repository of high-throughput genomic data. Two MI datasets, namely, GSE48060 and GSE83500, were compiled, and GSE19188, representing non-small cell lung cancer, was included in the study. “GEOquery,” a bioinformatics R tool, was used to obtain all the raw expression matrices (Davis and Meltzer, 2007). Table 1 shows a comprehensive description of the training group’s and external validation group’s demographic characteristics. In order to ensure the consistency of the data, the matching probe values of genes with multiple probes were processed using specific methods, a random value retained for each gene, with redundant values removed. Datasets underwent normalization involving exclusion of missing values and automated log2-transformation of non-log-transformed expression values. The overall analytical workflow is depicted in Figure 1.

**TABLE 1:** Details of the 3 GEO datasets.

Dataset	Platform	Control sample size	MI or NSCLC sample size	Sources	Application
GSE19188	GPL570	65	91	Lung tissue	DEGs, machine learning and WGNA
GSE48060	GPL570	21	31	Peripheral blood	DEGs, machine learning and WGNA
GSE83500	GPL13667	17	20	Frozen aortic tissues	Validation of hub genes

### 2.2 Identification of differentially expressed genes (DEGs)

DEGs were identified using the limma package (Ritchie et al., 2015), which is based on a robust linear modeling framework. The genes selected from the MI and NSCLC datasets, which satisfied the criteria of an adjusted p < 0.05 and |log_2_FC| > 0.5, were considered DEGs. Heatmaps were utilized to visualize the expression pattern of the 50 most significant genes in GSE19188 and GSE48060. The common genes, which were selected by different screening approaches, were determined by means of a Venn diagram, which was created using an online tool (http://www.bioinformatics.com.cn).

### 2.3 Weighted gene correlation network analysis (WGCNA)

WGCNA was used to identify disease-associated co-expression modules. Initially, the top 5,000 genes exhibiting the highest median absolute deviation (MAD) were selected (Langfelder and Horvath, 2008). Sample clustering and outlier removal were performed using the goodSamplesGenes function. We applied the conventional one-step WGCNA methodology to build a topological overlap matrix (TOM), tuning the soft-thresholding power to ensure a dependable adjacency matrix. A signed network was assumed for adjacency calculation to preserve the direction of gene co-expression correlations. Modules with very comparable eigengene expression patterns were further combined using a mergeCutHeight threshold of 0.3 to guarantee biologically significant clustering. The “disease” condition was identified as the primary phenotypic characteristic of interest in the ensuing association studies. Each gene’s module membership (MM), which measures how much each gene contributes to its own module, was then calculated by evaluating its correlation with the representative module eigengene, while gene significance (GS) represented correlations between genes and external traits. Hub genes showing maximal association with disease status were retained for downstream analyses. In this study, β values of 5 and 6 were selected for GSE48060 and GSE19188, respectively.

### 2.4 Machine learning selection of hub genes

Hub genes associated with MI and NSCLC were identified using an integrated machine-learning framework. To leverage the model’s robust ability to handle complex, high-dimensional datasets by maximizing the decision margin between classification boundaries, a support vector machine (SVM) classifier was first built and trained using specific feature gene subsets (Sanz et al., 2018). Feature space was then refined using SVM-recursive feature elimination (SVM-RFE), iteratively removing low-impact features to enhance diagnostic precision. Further variable selection employed LASSO regression (glmnet v4.1.8) with regularization to mitigate overfitting. The 1-standard error criterion (1-SE) guided optimal feature retention. Finally, random forest analysis ranked gene importance, with genes exceeding a relative importance score of 0.25 retained. In our study, we first used SVM-RFE to get the hub gene before using LASSO and random forest methods to determine the final target gene.

### 2.5 Enrichment analysis

KEGG pathway and GO enrichment analyses (BP, CC, MF) were performed using the clusterProfiler R package (Yu et al., 2012).

### 2.6 Protein-protein interaction network construction

STRING (http://string-db.org) was employed to create a PPI network corresponding to genes shared by MI and NSCLC. To provide sufficient dependability, only interactions with a minimum confidence score of ≥0.4 were kept. In order to find gene clusters that are biologically meaningful, the interaction network that was produced served as the basis for further topological and functional investigations.

### 2.7 Immune infiltration analysis

In order to elucidate immune pathway involvement in MI development among NSCLC patients, immune infiltration profiles of MI and control samples were quantified using CIBERSORT (R package) (Li et al., 2024). Samples with deconvolution P > 0.05 were excluded. We evaluated the differential abundance of 22 immune cell types using the Wilcoxon rank-sum test.

### 2.8 Single gene GSEA analysis

Gene Set Enrichment Analysis (GSEA) was performed on the identified hub genes, which were pre-ranked according to their respective correlation coefficients, to extensively examine the potential biological and functional roles of the genes. The use of this analytical tool helped to gain further insights into the molecular pathways and mechanisms of the key genes. In addition, the process of single-gene GSEA was carried out independently by making use of the meticulously curated gene sets present in the Molecular Signatures Database (MSigDB) along with the transcriptional expression profiles obtained from the GSE83500 dataset. In order to facilitate the standardized functional comparison, the KEGG pathway collection was designated as the background reference set in all the evaluation analyses, thus enabling the accurate mapping of the enriched functional categories to existing biological pathways. Those pathways, in which the adjusted P-value was found to be less than 0.05 in combination with a value of |NES| being greater than 0.5, were considered to have exhibited statistically significant associations, thus fulfilling the criteria for biological relevance.

### 2.9 *In vivo* experimental validation

Male C57BL/6 mice aged 6–8 weeks, including WT and STEAP4-KO mice, were used for *in vivo* validation. STEAP4-KO mice were obtained from Jiangsu Qinglongshan Biotechnology Co., Ltd. (China), and *in vivo* experiments were conducted to investigate the biological role of STEAP4 in the MI–NSCLC comorbidity model, thereby providing experimental validation for the bioinformatic findings. All the mice were kept under strictly controlled conditions in a certified specific pathogen-free (SPF) animal facility. The environmental conditions, including the photoperiod (a strict 12-h light-dark cycle), the temperature (a precisely controlled ambient temperature of 22 °C ± 2 °C), and the relative humidity level (a stable level of 50% ± 5%), were rigorously controlled and monitored during the experimental period. The mice had access to food and water *ad libitum*, with sterilized water and standard laboratory food. This ensured the nutritional and hydration requirements of the mice while keeping external variables under control. The Shanghai Tenth People’s Hospital Animal Ethics Committee granted ethical clearance for the complete experimental protocol (clearance No. SHDSYY-2024-0015), and all procedures closely followed *Guidelines for the Ethical Review of Laboratory Animal Welfare*. The mice were divided into seven groups at random: sham (Sham), MI, lung cancer (NSCLC), comorbidity model (MI + NSCLC), and STEAP4 knockout comorbidity model (STEAP4-KO-MI + NSCLC), the negative control siRNA comorbidity group (Si-NC-MI + NSCLC), and the STEAP4 siRNA intervention comorbidity group (Si-STEAP4-MI + NSCLC). The model was constructed by permanently ligating LAD coronary artery to induce MI, and lung cancer (NSCLC) was modeled by orthotopically implanting lung cancer cells into the pulmonary tissue, specifically 1 × 10^6^ Lewis lung carcinoma (LLC) cells (mouse origin, syngeneic to C57BL/6) suspended in 50 μL PBS were injected into the left lung parenchyma. The cells were maintained *in vitro* (passages 5–8) prior to implantation. Tumor engraftment was confirmed at euthanasia by macroscopic inspection and HE staining. The comorbidity model was established by implanting LLC cells 3 days after MI surgery. The siRNAs (negative control and STEAP4-specific) were commercially purchased (RiboBio Co., Ltd., Guangzhou, China). For siRNA intervention groups (Si-NC-MI + NSCLC and Si-STEAP4-MI + NSCLC), intravenous tail injections (6 mg/kg) were administered twice per week for three consecutive weeks, beginning on the third day post-implantation to allow tumor engraftment and avoid interference with the acute post-MI inflammatory phase. Mice were euthanized by inhalation of excessive isoflurane, with cessation of respiration and heartbeat defined as the endpoint. Cardiac and pulmonary tissues, as well as blood samples, were collected immediately for subsequent analyses.

### 2.10 Echocardiographic assessment of left ventricular structure and function

Mice were anesthetized by inhalation of 1% isoflurane, and anterior thoracic hair was removed. The Vevo 2,100 high-resolution small-animal ultrasound imaging equipment (VisualSonics, Canada) with a high-frequency MS-400 probe (30 MHz) was used to assess left ventricular anatomy and functional performance. To ensure an accurate assessment of both systolic and diastolic cardiac function, the left ventricular ejection fraction (LVEF) of each mouse was determined by measuring a minimum of three stable cardiac cycles using M-mode echocardiography.

### 2.11 Hematoxylin and eosin (HE) staining

The freshly isolated myocardial tissues and pulmonary tumor samples were meticulously harvested and immediately subjected to thorough washing with cold phosphate-buffered saline solution (PBS). Tissues were washed with cold PBS to remove surface blood and cellular debris, which might have interfered with the histological and molecular analysis conducted on the samples. All the cleaned tissue specimens were immersed in 4% freshly prepared paraformaldehyde solution and stored at 4 °C for an entire 24-h period. The fixing of the tissues helped to ensure that the cellular structures and tissues were properly preserved, thus guaranteeing optimal stability and quality. After fixation, tissues were dehydrated through graded ethanol solutions and embedded in paraffin. Hematoxylin and eosin (HE) staining was then used for the preparation and staining of thin slices, which were 5 μm thick. Sections were examined using a Nikon Eclipse E100 optical microscope.

### 2.12 Masson’s trichrome staining

For histological examination, the tissue blocks embedded in paraffin were sectioned into small pieces, with the final section thickness being approximately 4 μm. Masson’s trichrome staining was performed using a commercial Masson’s trichrome staining kit (Agilent/Dako) following the manufacturer’s protocol. This ensured the use of uniform staining techniques, thus minimizing human error in the entire procedure. The sections were then scanned using a digital slide scanner, i.e., Aperio XT, USA.

### 2.13 ELISA

We collected whole blood samples and stored them at room temperature for about two hours to ensure that the samples had clotted completely. After the samples had clotted, they were centrifuged at 1,000 × g for 15 min at 4 °C, and clear serum samples were obtained. For each sample, 50 μL of the serum samples and 50 μL of the diluent were thoroughly mixed. The absorbance values were then measured rigorously, as guided by the operating instructions in the manuals for the ELISA kits. The concentration of TNF-α and IL-6 in each of the experimental groups was calculated. The ELISA kits used were from Neobioscience Technology Co., Ltd. (Shenzhen, China) with catalog numbers EMC004 (for IL-6) and EMC102a (for TNF-α), and all procedures were performed according to the manufacturer’s instructions.

### 2.14 Western blot (WB) analysis

For total protein extraction, 10 mg of myocardium or tumor tissue from each group was homogenized in RIPA buffer containing PMSF (100:1). Protein concentration was quantified using a BCA assay. Equal amounts (20 μg/lane) of protein were mixed with 5× loading buffer, denatured, and separated by SDS-PAGE. After transfer to PVDF membranes, the membranes were blocked with 5% non-fat milk in TBST for 1 h at room temperature, then incubated overnight at 4 °C with primary antibodies: anti-STEAP4 (Proteintech, 1:1000) and anti-β-actin (Proteintech, 1:5000). After washing with TBST, membranes were incubated with HRP-conjugated secondary antibody (Proteintech, 1:5000) for 2 h at room temperature. Protein bands were visualized using enhanced chemiluminescence and quantified with ImageJ software.

### 2.15 Quantitative real-time PCR analysis

Total RNA was extracted from heart and lung tissues using TRIzol reagent. Reverse transcription was performed using PrimeScript™ RT Master Mix (TaKaRa, Cat# RR036A) with 1 µg of total RNA in a 20 µL reaction. qPCR was performed using TB Green® Premix Ex Taq™ II (TaKaRa, Cat# RR820A) on a QuantStudio 5 Real-Time PCR System. The primer sequences for target genes (Ly6g, S100a8, Cd8a, Gzmb) and β-actin are listed in Table 2. The PCR protocol was: 95 °C for 3 min, followed by 40 cycles of 95 °C for 10 s and 56 °C for 30 s. Melting curve analysis was performed to confirm product specificity. All reactions were performed in triplicate, and relative expression was calculated using the 2^−ΔΔCt^ method with β-actin as the internal control.

**TABLE 2:** Primer sequence used for mRNA RT-qPCR.

Gene	Sequences (5′–3′)
*Ly6G*	F: GACTTCCTGCAACACAACTACC R: ACAGCATTACCAGTGATCTCAGT
*S100a8*	F: CAAGGAAATCACCATGCCCTCTA R: ACCATCGCAAGGAACTCCTCGA
*Cd8a*	F: ACTACCAAGCCAGTGCTGCGAA R: ATCACAGGCGAAGTCCAATCCG
*Gzmb*	F: CAGGAGAAGACCCAGCAAGTCA R: CTCACAGCTCTAGTCCTCTTGG
*β-actin*	F: GTCAGGTCATCACTATCGGCAAT R: AGAGGTCTTTACGGATGTCAACGT

### 2.16 Statistical analysis

Statistical analysis was conducted based on the properties and features of the collected data. In addition, extensive bioinformatics analysis, including preprocessing, statistical evaluation, and visualization, was conducted using the R software version 4.3.0. We compared two independent groups by applying either Student’s t-test or the Wilcoxon rank-sum test, based on whether the data met parametric assumptions. We applied Spearman correlation analysis to explore the relationship between immune cell distribution and the expression levels of representative genes, thereby uncovering potential immunological and molecular crosstalk in the disease microenvironment. We expressed animal experimental data as mean ± standard deviation (Mean ± SD) and conducted statistical analyses and figure generation with GraphPad Prism 9.0. Group differences were assessed using one-way analysis of variance (ANOVA), followed by Tukey’s *post hoc* test for multiple comparisons when significance was detected. A P value <0.05 was considered statistically significant.

## 3 Results

### 3.1 Identification of DEGs and WGCNA modules in MI

**FIGURE 2:**
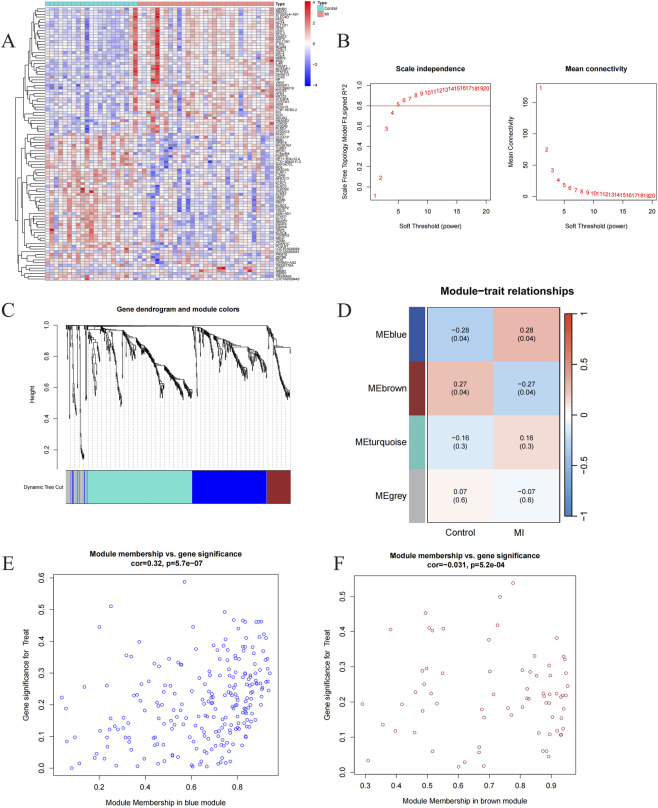
The construction of weighted gene co-expression network and identification of DEGs in MI. **(A)** Heatmap depicting the most DEGs following differential analysis of the grouped GSE48060 dataset. Genes with considerably lower expression levels are shown by blue dots, whereas genes with increased expression in MI samples are indicated by red dots. MI: myocardial infarction. **(B)** A robust construction of the MI co-expression network was made possible by the optimal soft-thresholding power of β = 5, which was established based on analyses of scale independence and mean connectivity. **(C)** A hierarchical clustering dendrogram of genes is shown, with different co-expression modules represented by color-coded blocks beneath the tree structure. **(D)** A heatmap that illustrates the correlations between module eigengenes and clinical traits is displayed. Strong correlation coefficients and statistical significance led to the selection of the brown and blue modules for additional analysis. **(E,F)** Genes within the brown and blue modules are shown in scatter plots that show the relationship between GS and MM, indicating the extent to which core genes contribute to MI-related module functions.

By comparing MI samples with non-MI control samples using gene expression profiling of the GSE48060 dataset, a total of 4,133 DEGs were identified. The top 100 DEGs were visualized using a heatmap to illustrate their expression patterns, since these DEGs showed significant changes in expression between the two situations (Figure 2A). WGCNA was used to look at coordinated gene expression changes associated with MI in more detail. Following the evaluation of several possible soft-thresholding powers, β = 5 was determined to be the best value. This resulted in a scale-free network structure with minimal mean connectivity and a high signed R2 topology fit, which is in line with the properties of a biologically realistic network (Figure 2B). The clustering dendrogram identified four major co-expression modules based on gene expression similarity: the blue module (MEblue), brown module (MEbrown), turquoise module (MEturquoise), and grey module (MEgrey). The grey module contained genes that were not included in any particular cluster (Figure 2C). Our analysis of module eigengenes and clinical features indicated that the blue module was positively correlated with the MI phenotype (cor = 0.28, p = 0.04), whereas the brown module correlated positively (cor = 0.27, p = 0.04). Although the absolute correlation values are below 0.5, both reached statistical significance (p < 0.05), and the biological relevance of these modules was further supported by subsequent machine learning and experimental validation. These results imply that both modules could have a functional role in the pathophysiology of MI (Figure 2D). Subsequent intramodular connectivity analysis showed that the blue module’s central hub genes were strongly associated with MI, as evidenced by the moderately positive correlation between the MM values and GS scores (cor = 0.32, p = 5.7e-07). Conversely, we observed that the brown module exhibited a modest MM–GS correlation (cor = −0.031, p = 5.2e−04), implying increased variability among its gene associations (Figures 2E,F). Notably, all four final core genes (STEAP4, HP, TREM1, CCR2) were derived from the blue module, not from the brown module. Therefore, the weak correlation in the brown module does not affect the identification or validation of our core biomarkers.

### 3.2 Identification of DEGs and WGCNA modules in NSCLC

**FIGURE 3:**
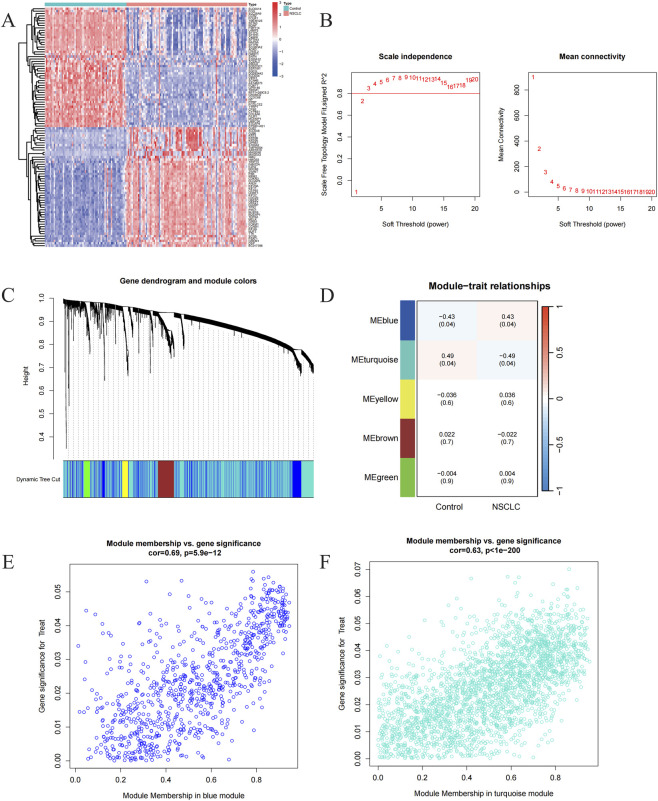
The construction of weighted gene co-expression network and identification of DEGs in NSCLC. **(A)** Heatmap depicting 50 most DEGs following differential analysis of the grouped GSE19188 dataset. Red markers show upregulated genes, while blue markers show downregulated genes. **(B)** A robust NSCLC co-expression network was ensured by selecting a soft-thresholding power β = 6 based on an evaluation of scale-free topology and connectivity metrics. **(C)** A gene clustering dendrogram is displayed, with co-expression modules identified by color assignments. **(D)** The heatmap of module–trait correlations identifies the blue and turquoise modules as biologically relevant because of their statistically significant correlations with NSCLC characteristics. **(E,F)** Scatter plots show the relationships between MM and GS for genes in the blue and turquoise modules, confirming strong intramodular connectivity.

In the GSE19188 dataset, differential expression analysis identified a total of 2,038 DEGs between NSCLC samples and normal controls. The top 100 genes with the most significant expression changes were visualized via a heatmap (Figure 3A). The NSCLC data set was further subjected to WGCNA to gain a deeper understanding of the co-expression gene patterns in NSCLC. This enabled the creation of gene modules, which grouped genes together in terms of highly correlated expression levels, thus providing a systems view of the transcriptional architecture of NSCLC. After soft-threshold power selection, β = 6 was determined as the optimal parameter, producing a network with desirable scale-free topological characteristics (Figure 3B). Gene clustering analysis identified five major co-expression modules: blue (MEblue), turquoise (MEturquoise), yellow (MEyellow), brown (MEbrown), and green (MEgreen), along with a grey module containing unclassified genes (Figure 3C). According to module–trait correlation analysis, the turquoise module showed a positive association (cor = 0.49, p = 0.04) with the NSCLC phenotype, whereas the blue module showed a negative correlation (cor = −0.43, p = 0.04). Although these correlation coefficients are slightly below 0.5, both were statistically significant (p < 0.05), and the importance of these modules was independently confirmed by multiple machine learning algorithms and *in vivo* experiments. These findings suggest that both modules may be involved in NSCLC-related molecular processes (Figure 3D). The blue module (cor = 0.69, p = 5.9e−12) and the turquoise module (cor = 0.63, p < 1e−200) both showed a substantial and positive correlation between MM and GS, according to further examination of the internal modular structure. Genes located in the center of these modules may serve as important regulatory drivers of NSCLC pathology, according to this substantial MM–GS association (Figures 3E,F). This result indicated that hub genes within these modules were highly relevant to the NSCLC phenotype. Based on these analyses, the blue module (288 genes) and turquoise module (567 genes) were selected as the key NSCLC-related modules, comprising a total of 855 genes for subsequent research. The remaining modules—including the grey (254 genes), green (84 genes), and yellow (85 genes) modules—were excluded from further analysis.

### 3.3 Identification of shared genes between MI and NSCLC

**FIGURE 4:**
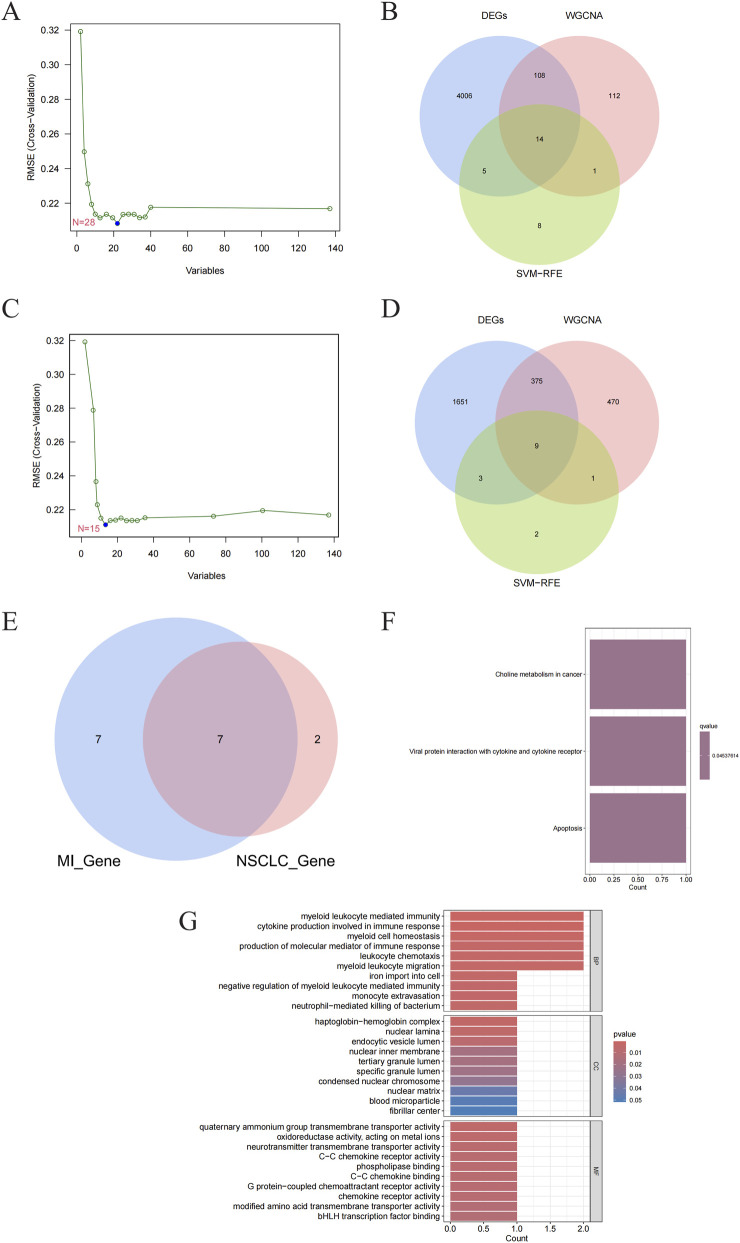
Identification of genes and functional enrichment associated with both MI and NSCLC. **(A)** Performance Plot of Recursive Feature Elimination for GSE48060. The x-axis shows how many genes are in the model, and the y-axis shows the likelihood of an error. **(B)** A Venn diagram shows the overlap among genes selected by SVM-RFE, DEGs, and WGCNA for MI, yielding 14 MI-characteristic genes. **(C)** The performance plot of recursive feature elimination for the GSE19188 dataset is shown, where the minimal error probability was achieved at N = 15. **(D)** A Venn diagram shows the intersection of SVM-RFE–selected genes, DEGs, and WGCNA modules for NSCLC, identifying 9 genes characteristic of NSCLC. **(E)** The final Venn diagram shows the intersection of the 14 MI-related genes and the 9 NSCLC-related genes, identifying a subset of genes exhibiting shared molecular features. **(F,G)** Functional enrichment analysis (KEGG and GO) of the dual-characteristic genes.

To systematically identify the key genes underlying the comorbidity between MI and NSCLC, a multi-stage screening strategy was implemented. The SVM–recursive feature elimination (SVM-RFE) approach was separately applied to the DEGs of MI and NSCLC in order to find genes simultaneously involved in both illnesses. Iterative ranking and the removal of less relevant genes were made possible by this machine-learning-based feature selection process, which finally isolated the most informative and disease-related shared gene signatures. In the MI dataset (GSE48060), 28 feature genes were identified, whereas 15 feature genes were extracted from the NSCLC dataset (GSE19188) (Figures 4A,C). Subsequently, an intersection analysis was conducted integrating the results from DEG analysis, WGCNA module identification, and SVM-RFE screening. In the MI dataset, 14 key genes were jointly identified by all three approaches: ADM, DSC2, KCNJ15, LMNB1, NFIL3, PI3, ACSL1, TDRD9, CCR2, SLC22A4, HP, STEAP4, NCAPG2, and TREM1 (Figure 4B). In the NSCLC dataset, 9 key genes were consistently identified across the three methods: NCAPG2, LMNB1, SLC22A4, TREM1, STEAP4, HP, CCR2, GRK5, and BCL3 (Figure 4D). Cross-comparison of the two datasets identified seven overlapping genes between MI and NSCLC, including CCR2, LMNB1, SLC22A4, HP, STEAP4, NCAPG2, and TREM1 (Figure 4E). These genes exhibited significant expression changes and module characteristics in both diseases and were regarded as potential molecular bridges connecting MI and NSCLC comorbidity mechanisms.

### 3.4 Enrichment analysis of shared genes

To further elucidate the biological functions of the 7 shared genes (CCR2, LMNB1, SLC22A4, HP, STEAP4, NCAPG2, and TREM1), comprehensive enrichment analyses were performed, including Kyoto Encyclopedia of Genes and Genomes (KEGG) and GO analyses. KEGG pathway enrichment analysis identified that genes predominantly cluster in three key pathways (Figure 4F), including apoptosis, viral proteins modulating cytokine and receptor networks, and cancer-related choline metabolism. These enrichment patterns indicate that the shared genes significantly contribute to comorbidity biology by modulating immune signaling pathways, altering metabolic programs, and regulating mechanisms of programmed cell death. The complex biological characteristics of these genes were further clarified by Gene Ontology (GO) enrichment analysis (Figure 4G). Enriched GO terms were mainly associated with immune-related processes at the biological process (BP) level, including cytokine generation, myeloid cell homeostasis maintenance, and myeloid leukocyte-mediated immunity. Endocytic vesicle lumen, nuclear lamina, and the hemoglobin heme complex are some of the structures identified as being enriched at the cellular component level. This points to the particular subcellular location and functions of the genes. The analysis of the molecular function level identified the main functions of the genes as being oxidoreductases, transport of neurotransmitters across membranes, and transmembrane transport of quaternary ammonium. Collectively, the enrichment results for the shared genes indicate that the 7 shared genes could be involved in the comorbidity between MI and NSCLC through various mechanisms, including the coordination of the immune response, maintenance of the integrity of the cell structures, and the regulation of transmembrane transport, which provide the functional evidence for the shared genes.

### 3.5 Screening and validation of core genes

**FIGURE 5:**
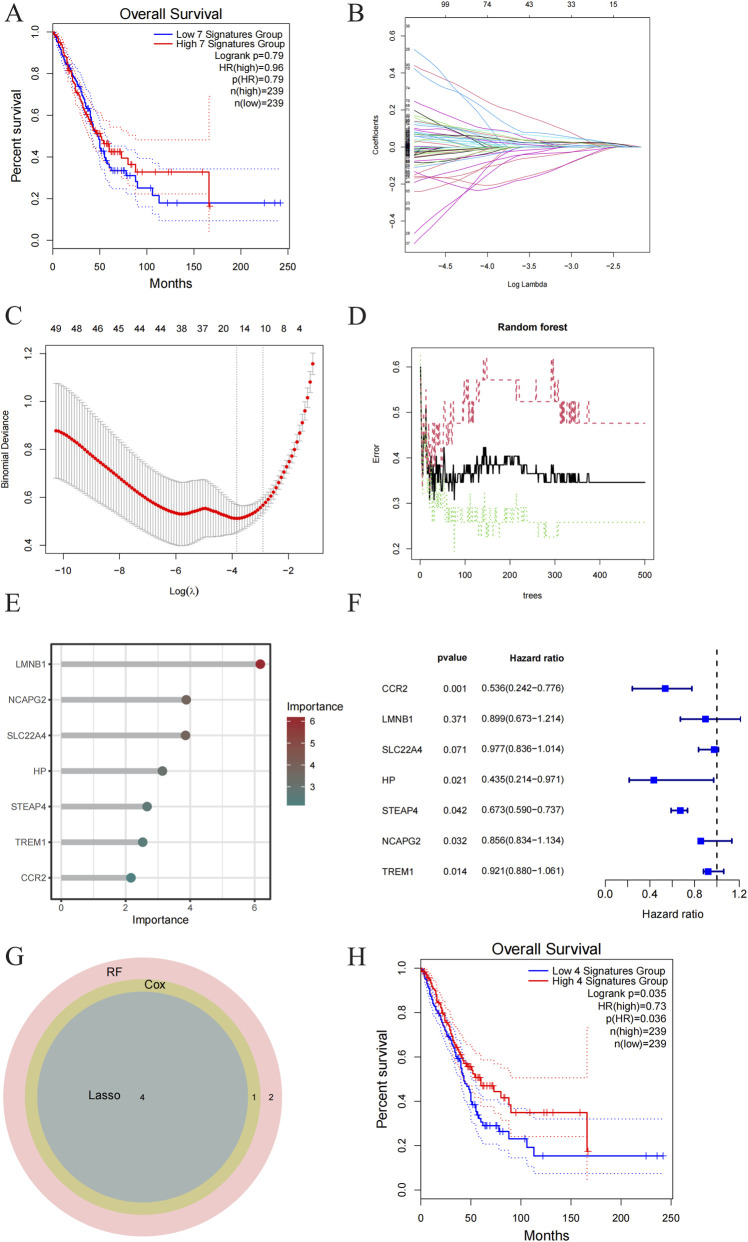
Identification of candidate hub genes via machine learning. **(A)** Analyzed with the KM curves of the seven genes in TCGA-LUAD in Figure 4, there was a trend that the lower the expression the worse the prognosis of the patients, but P = 0.79, which was not statistically significant. **(B)** The LASSO coefficient profiles of candidate genes are displayed, demonstrating how coefficients shrink across different λ values. **(C)** The partial likelihood deviance curve is presented, indicating the optimal λ for LASSO regression. **(D)** Random forest error plot. **(E)** The MeanDecreaseGini index for the seven genes is displayed, emphasizing the seven genes with MeanDecreaseGini >1.0 chosen by the random forest algorithm as highly significant features **(F)** Multifactorial Cox analysis of forest plots associated with MI yielded six genes **(G)** RF, Lasso and multifactorial Cox results Wayne plots were obtained for our target gene: TREM1, HP, STEAP4, CCR2 **(H)** The KM curves of the four genes in TCGA-LUAD were analyzed with a trend of the lower the expression the worse the prognosis of the patients, which was consistent with the A graph and was statistically significant with P = 0.036.

To determine the prognostically most significant of the 7 core biomarkers identified within the overlapping genes, a multi-machine-learning-algorithm-and-survival-analysis strategy was employed. For the TCGA-LUAD cohort, the combined expression of the 7 selected genes was not found to hold statistical significance as a prognostic marker (Log-rank p = 0.79; HR = 0.96; p value for HR = 0.79), as assessed by Kaplan-Meier analysis. This indicates that the overall predictive power of these genes may be limited, although a trend towards a poor prognosis was observed in the low-expression group (Figure 5A). Subsequently, sophisticated machine learning techniques were employed for further selection. The least absolute shrinkage and selection operator (LASSO) regression model was used to further narrow the selection; in order to avoid overfitting, cross-validation was used to determine the ideal λ value. Genes above the threshold (>1.0) were deemed highly informative by the random forest method, which simultaneously assessed gene relevance using the MeanDecreaseGini score (Figures 5B–E). Furthermore, six genes were shown to have significant relationships with the prognosis of MI using multivariate Cox regression analysis, enhancing their significance as disease-related biomarkers (Figure 5F). A Venn diagram integrating the results from the three methods revealed four overlapping genes—TREM1, HP, STEAP4, and CCR2—which were therefore established as the core biomarkers of MI–NSCLC comorbidity (Figure 5G). To validate the prognostic significance of these four core genes, an additional Kaplan–Meier analysis was conducted using the TCGA-LUAD dataset. The combined expression level of the four genes showed a significant correlation with overall survival (Figure 5H). In comparison with the high-expression group, the low-expression group showed significantly poorer survival than the high-expression group (Log-rank p = 0.035; HR = 0.73, p (HR) = 0.036). This finding was consistent with the previous trend and reached statistical significance, which validated the potential predictive value of the four genes for the prognosis of NSCLC, especially in the context of the possible co-occurrence of MI.

### 3.6 Evaluation of diagnostic performance and construction of the nomogram for core genes

**FIGURE 6:**
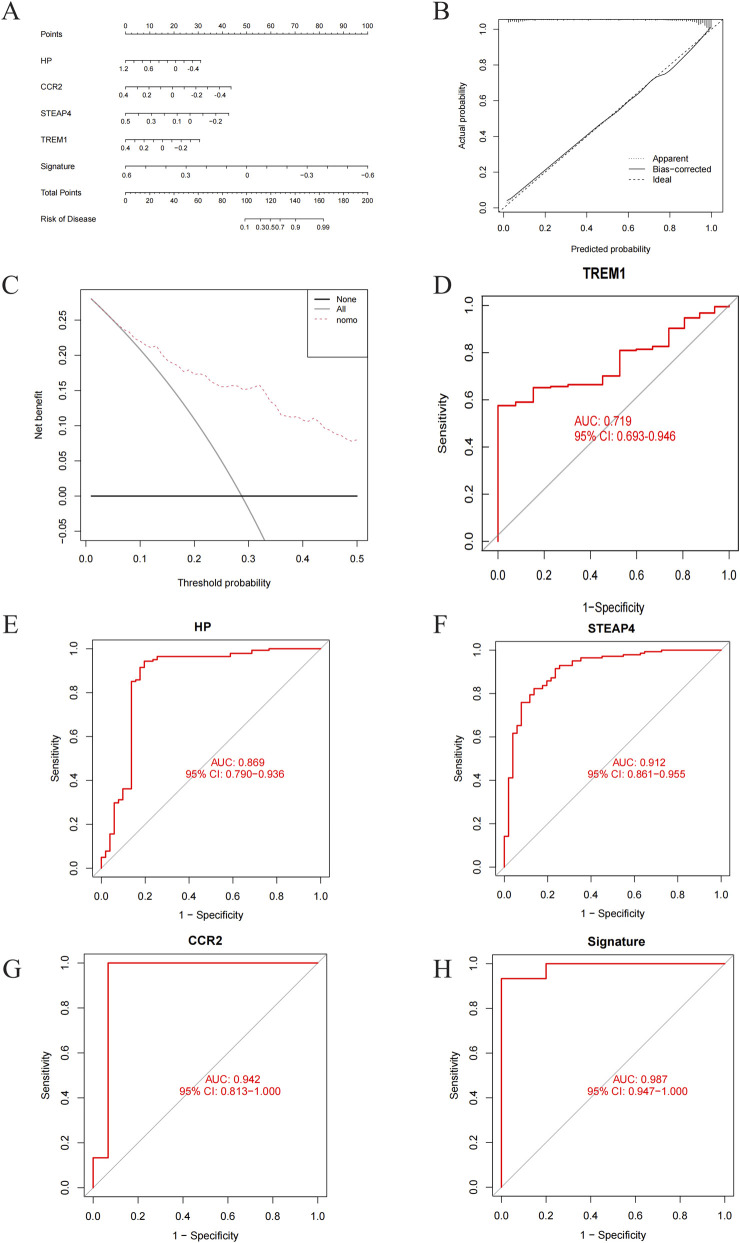
Assessment and validation of hub genes value and nomogram construction. **(A)** A nomogram was constructed in the MI dataset based on four genes: TREM1, HP, STEAP4, and CCR2. **(B,C)** Show nomogram calibration curve and DCA curve **(D–H)**The ROC curves of TREM1, HP, STEAP4, CCR2 and Signature are shown separately.

Predictive modeling and validation analyses were performed to evaluate the diagnostic and prognostic utility of four core genes (TREM1, HP, STEAP4, and CCR2). A nomogram integrating the expression levels of these genes was developed using the MI dataset. Decision curve analysis demonstrated notable clinical net benefits across a wide range of relevant threshold probabilities, and calibration curves indicated strong agreement between predicted risks and observed outcomes (Figures 6A–C). ROC curve analysis was subsequently used to assess the diagnostic performance of individual genes. Particularly good diagnostic accuracy for STEAP4 and CCR2 was shown by the area under the curve (AUC) values of 0.719 (95% CI: 0.693–0.946) for TREM1, 0.869 (95% CI: 0.790–0.936) for HP, 0.912 (95% CI: 0.861–0.955) for STEAP4, and 0.942 (95% CI: 0.813–1.000) for CCR2 (Figures 6D–G). Most remarkably, the integrated diagnostic model outperformed any single-gene marker by merging the four genes into a multi-marker signature, achieving an outstanding AUC of 0.987 (95% CI: 0.947–1.000) (Figure 6H). These findings collectively verified the high diagnostic reliability of TREM1, HP, STEAP4, and CCR2 as potential biomarkers for MI–NSCLC comorbidity, with the four-gene integrated model exhibiting superior accuracy and offering a robust foundation for clinical translation.

### 3.7 Immune cell infiltration and correlation between core genes and immune cell subtypes

**FIGURE 7:**
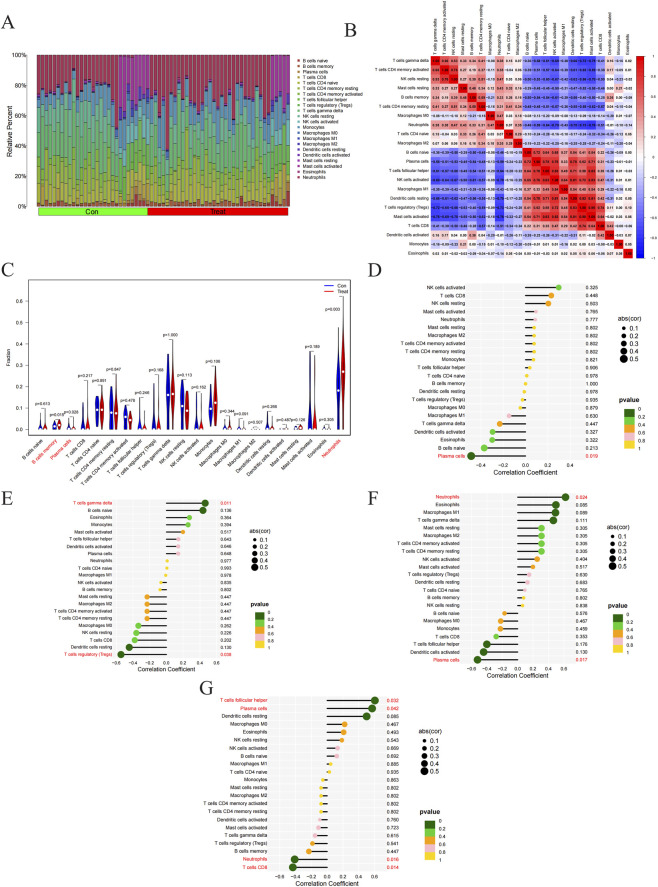
Immune cell infiltration and correlation analysis. **(A)** Immuno-infiltration analysis of GSE83500 was performed. **(B)** Heatmaps were plotted. **(C)** Immunocellular difference analysis was done according to control and MI groups using violin diagrams. The relationships between four key genes—TREM1, HP, STEAP4, and CCR2—and several immune cell subpopulations are shown in **(D–G)** lollipop plots. Each figure illustrates the distinct immune regulatory patterns connected to each gene by visualizing the correlation’s amplitude and direction.

A detailed analysis of immune cell infiltration was carried out based on the GSE83500 dataset to explore the immunological basis of the development of MI and to further elucidate the potential relationship between the essential genes and immune microenvironment regulation. The analysis showed obvious differences between the immune microenvironment of MI patients and healthy individuals. The MI samples had more memory B cells and neutrophils, but fewer plasma cells proportionally, compared with the controls (Figures 7A–C). The functional immunological significance of the key genes identified above was further explored by evaluating their associations with specific immune cell types (Figures 7D–G). Plasma cell abundance and TREM1 expression showed a strong negative association (P = 0.019), suggesting that increased TREM1 may reflect or repress decreased plasma cell activity. Conversely, HP expression showed a significant negative correlation with regulatory T cells (Tregs) (P = 0.038) and a strong positive correlation with γδ T-cell infiltration (P = 0.011), indicating that it may have a dual role in promoting inflammatory responses and undermining immune suppression; STEAP4 expression was positively correlated with neutrophil abundance (P = 0.024) and negatively correlated with plasma cells (P = 0.017); while CCR2 exhibited positive correlations with follicular helper T cells (P = 0.032) and neutrophils (P = 0.016), but negative correlations with plasma cells (P = 0.042) and CD8^+^ T cells (P = 0.014). These observations indicate that TREM1, HP, STEAP4, and CCR2 may play crucial roles in the comorbidity mechanism of MI and NSCLC by modulating the infiltration and function of specific immune cell populations, particularly neutrophils, plasma cells, and T-cell subsets.

### 3.8 *In vivo* validation of the critical role of STEAP4 in MI–NSCLC comorbidity

**FIGURE 8:**
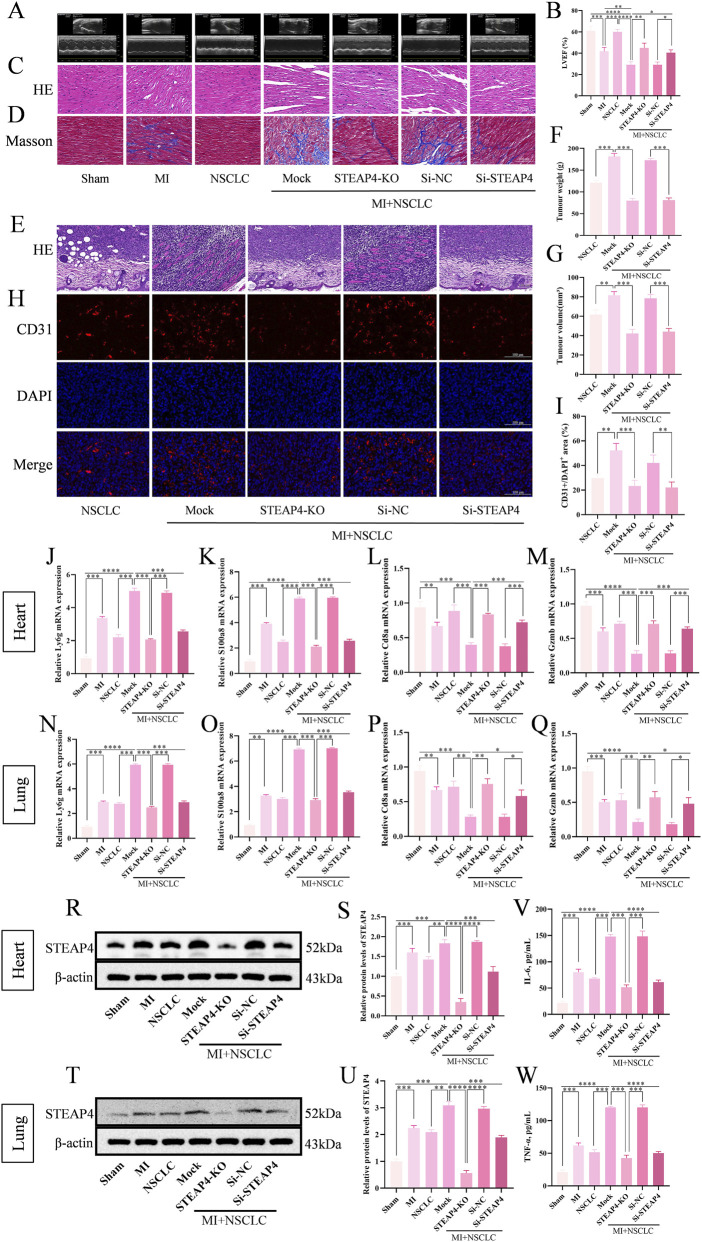
*In vivo* validation of the critical role of STEAP4 in MI–NSCLC comorbidity. **(A,B)** Quantitative measurements of left ventricular ejection fraction (LVEF) and representative echocardiograms are displayed for each experimental group, demonstrating differences in cardiac function. **(C,D)** Quantitative evaluations of infarct area and fibrosis are presented along with representative histological images of cardiac tissue sections stained with hematoxylin and eosin (HE) and Masson’s trichrome. **(E–G)** Quantitative analyses of tumor burden, including tumor weight and volume, are presented along with representative HE-stained images of lung tissue sections. **(H,I)** Representative immunofluorescence images of CD31 in tumor tissues and quantitative analysis of microvessel density. **(J–Q)** qRT-PCR analysis of immune cell markers in cardiac and pulmonary tissues: Expression levels of neutrophil markers Ly6g and S100a8, CD8^+^ T cell marker Cd8a, and functional effector Gzmb in heart and lung tissues (n = 6). **(R–U)** Western blot images and corresponding quantitative analyses show the expression levels of the STEAP4 protein in both myocardial and pulmonary tissues across groups (n = 3). **(V,W)** Serum IL-6 and TNF-α concentrations measured by ELISA are displayed (n = 6). The mean ± SD is used to represent the data. Statistical comparisons among groups were performed using one-way ANOVA with Tukey’s *post hoc* test. The following symbols indicate statistical significance: *p < 0.05, **p < 0.01, ***p < 0.001, and ****p < 0.0001.

Mice models of MI, NSCLC, and their combined comorbidities were effectively created in order to experimentally confirm the functional significance of STEAP4 *in vivo*. Mice in the comorbidity (MI + NSCLC) group had a significantly lower LVEF than the sham-operated controls, indicating a significant deterioration of cardiac systolic function, according to echocardiographic examination (Figures 8A,B). The degree of fibrosis and the amount of infarcted cardiac tissue in the comorbidity group were both significantly greater than those in the MI-only or NSCLC-only groups, according to complementary histopathological evaluations, suggesting significant structural damage and pathological remodeling (Figures 8C,D). In terms of tumor phenotype, hematoxylin–eosin staining of lung tissues showed blurred boundaries between tumor and adjacent normal tissues, with enhanced invasiveness and significantly increased tumor mass and volume in the comorbidity group compared with the NSCLC-only group, suggesting markedly elevated tumor burden (Figures 8E–G). CD31 immunofluorescence staining revealed a notable increase in intratumoral microvessel density in the comorbidity group, indicating enhanced angiogenic capability and a higher degree of malignancy (Figures 8H,I). Mechanistic investigation demonstrated that comorbidity induced significant immune microenvironmental disruption. qRT-PCR analysis of immune cell markers revealed significantly elevated expression levels of the neutrophil markers Ly6g and S100a8 in both cardiac and pulmonary tissues of MI + NSCLC mice, whereas the expression of CD8^+^ T cell marker Cd8a and functional effector Gzmb were substantially reduced, indicating enhanced neutrophilic inflammation and impaired cytotoxic T cell function in the comorbidity group (Figures 8J–Q). Western blot studies demonstrated a substantial increase in STEAP4 protein expression at the molecular level in the comorbidity mice’s lung and heart tissues (Figures 8R–T–U). The idea of increased systemic inflammation in comorbid conditions was further supported by ELISA results, which showed that serum concentrations of the pro-inflammatory cytokines TNF-α and IL-6 were significantly higher in the comorbidity group than in the single-disease models (Figures 8V,W), indicating a close association between STEAP4 and systemic inflammatory activation. Notably, STEAP4 intervention reversed the comorbidity-associated phenotypes. In comparison with the MI + NSCLC group, the treatment groups with STEAP4-KO and Si-STEAP4 showed significant improvements in heart function, myocardial fibrosis, tumor growth, immune balance, and levels of inflammatory cytokines, while the control group with Si-NC did not show significant improvements. These findings further confirm that the reversal effects were specifically mediated by STEAP4 regulation. The results collectively indicate that STEAP4 is pivotal in the development of MI-NSCLC comorbidity by regulating inflammatory activation and immune homeostasis in the local and systemic microenvironments.

## 4 Discussion

NSCLC and MI remain two of the most common and deadly diseases in the world, causing widespread morbidity, disability, and mortality. In addition to affecting the course of the disease, the co-existence of NSCLC and MI poses great challenges in clinical care and therapeutic decisions, with intricate molecular mechanisms underlying the clinical comorbidity between them (Sun et al., 2021; Moledina et al., 2022; Jani et al., 2023). With the progress of precision medicine technology, epidemiological studies further demonstrated that significant associations exist between the two diseases, and patients with comorbidity generally show poor clinical outcomes and complex therapeutic responses. Based on the combination of bioinformatics and experimental validation, this study systematically identified and verified STEAP4, HP, TREM1, and CCR2 as key genes related to MI-NSCLC comorbidity and established a new theoretical basis for understanding the molecular basis of the two diseases.

At the functional level, the four genes form a multidimensional network. STEAP4, which is a metalloreductase, plays protective roles in inflammation and metabolic stress by regulating iron/copper metabolism and oxidative stress (Scarl et al., 2017; Yang et al., 2024). In the early ischemic phase of myocardial infarction, the expression of SLC40A1 (iron exporter) and STEAP4 is upregulated in cardiomyocytes, leading to increased iron efflux, mitochondrial dysfunction, oxidative stress, and apoptosis, which together cause heart failure (Feng et al., 2024). On the other hand, in tumor development, STEAP4 is associated with the regulation of cell growth, apoptosis, and angiogenesis (Li et al., 2021; Orfanou et al., 2021). Based on the aforementioned facts, it is proposed that STEAP4 acts as a link between post-MI cardiac remodeling and tumor microenvironment modification through the maintenance of cellular redox homeostasis. Of note, the three excluded genes (LMNB1, NCAPG2, SLC22A4) are mainly involved in nuclear structure, cell cycle, or drug transport, and their expression may not consistently correlate with survival in lung adenocarcinoma. In contrast, the four retained genes (STEAP4, HP, TREM1, CCR2) are all directly linked to inflammatory and immune pathways, which are more coherently associated with disease progression. This biological concordance likely explains why removing the three less-aligned genes improved the prognostic performance.

HP (Haptoglobin) is an important plasma glycoprotein that reduces oxidative damage by binding to free hemoglobin (Naryzny and Legina, 2021). During MI, free hemoglobin resulting from damaged red blood cells enhances oxidative stress, while HP reduces this effect by forming complexes with free hemoglobin that are removed by macrophages (Mehta and Reddy, 2015; MacKellar and Vigerust, 2016). It is interesting to note that previous studies have shown that the antioxidant function of the HP gene might be significantly affected by genetic variations. For example, individuals with the Hp2-2 genotype have been found to be at a much increased risk for cardiovascular diseases, especially for diabetic patients, and also possess lower antioxidant activity (Wan et al., 2021). In oncology, HP has been shown to be involved in tumor proliferation and metastasis, and it has also shown promise as a biomarker because of its aberrant glycosylation, and it further regulates angiogenesis and lymphangiogenesis (Mariño-Crespo et al., 2019; Krzyszczyk et al., 2020; Dalan and Liuh Ling, 2018). These findings have shown that HP has multifunctional roles in comorbidity regulation.

TREM1 is a critical activating receptor in myeloid cells and plays an important role in inflammatory immune responses (Panagopoulos et al., 2022). By enhancing the TLR and NLR signaling pathways, TREM1 augments the cytokine storm, characterized by the excessive production of pro-inflammatory cytokines (de Oliveira Matos et al., 2020; Labiano et al., 2022). In MI, TREM1 expression contributes to the infiltration of immune cells into the myocardium, which increases damage (Lin et al., 2023). In the NSCLC microenvironment, the expression of TREM1 in tumor-associated macrophages promotes tumor development (Nie et al., 2024). This dual behavior underscores the shared inflammation-driven nature of TREM1 in both diseases.

CCR2 (C–C Motif Chemokine Receptor 2) mediates CCL2 signaling and is crucial in inflammatory cell recruitment (Moadab et al., 2021). In the context of MI repair, CCR2-mediated monocyte recruitment exhibits dual effects: moderate recruitment facilitates tissue repair, while excessive activation induces fibrosis. Similarly, in NSCLC, the CCL2/CCR2 axis recruits immunosuppressive cells, promoting angiogenesis and metastasis (Weinberger et al., 2015). The strong association between CCR2 and the tumor immunosuppressive microenvironment thus provides an important molecular foundation linking the inflammatory pathways of both diseases.

Additional evidence connecting the core genes with immune microenvironmental remodeling was found by further investigation of immune infiltration. A pro-inflammatory axis was suggested by the positive association between CCR2 expression and increased neutrophil infiltration, while a possible inhibition of cytotoxic immune responses was indicated by the negative correlation with CD8^+^ T-cell abundance; STEAP4 showed a similar positive correlation with neutrophil infiltration, suggesting that these genes may act synergistically to coordinate myeloid and lymphoid immune responses. Given its pivotal position in the comorbidity mechanism, broad functional spectrum, and limited prior research, STEAP4 was selected for in-depth functional validation. Animal experiments confirmed that comorbid mice exhibited concurrent deterioration of cardiac function and increased tumor burden, accompanied by significant STEAP4 upregulation and immune microenvironmental imbalance. Notably, modest differences in the expression levels of the neutrophil markers Ly6g and S100a8, as well as STEAP4, were observed between the MI and NSCLC groups in pulmonary tissues, suggesting disease-specific inflammatory characteristics. Meanwhile, the reduced expression of the T-cell markers Cd8a and Gzmb indicated a potential remote immunomodulatory effect of MI on the pulmonary microenvironment. These changes were further amplified in the comorbidity group, indicating a synergistic effect of the two diseases. Conversely, the knockout or silencing of STEAP4 reversed all of these pathological phenotypes, thereby improving cardiac function and immune homeostasis while reducing fibrosis and tumor growth. These results not only establish the role of STEAP4 in MI-NSCLC comorbidity, but they also validate the predictions of bioinformatics, thus offering experimental aid in the development of a therapeutic target.

Despite these important findings, it should be noted that there were a number of limitations, including the possible influence of data set heterogeneity (specifically, the MI dataset GSE48060 is derived from peripheral blood whereas the NSCLC dataset GSE19188 is from lung tissue; this cross-tissue comparison may introduce bias), sample sizes, and the need for further *in vivo* validation for the mechanistic involvement of the newly found genes. Clinical cohort validation of these findings was not performed. In particular, no clinical cohort of MI-NSCLC comorbid patients was available to validate the expression or prognostic value of the identified hub genes. *In vitro* mechanistic experiments and analyses of neuroendocrine–immune interactions were also not performed. Furthermore, the varying expression patterns of these genes during different stages of the diseases are not sufficiently explored, which makes the clinical utility of these findings yet to be verified. We acknowledge that among the four core hub genes (STEAP4, HP, TREM1, CCR2), only STEAP4 was subjected to in-depth functional validation in this study. This prioritization was based on STEAP4 showing the highest diagnostic performance (AUC = 0.912) and being relatively understudied in cardio-oncology comorbidity compared to the other three. The specific roles of HP, TREM1, and CCR2 in MI-NSCLC comorbidity remain to be experimentally addressed. Additionally, we did not perform IHC or IF staining to determine the subcellular localization of STEAP4 in myocardial and tumor tissues, and the specific cell types in the heart and lung that predominantly express STEAP4 under comorbid conditions, as well as the functional implications of STEAP4 in different immune cell subsets, remain to be elucidated in future studies. Whether STEAP4 knockout exerts similar protective effects in MI-only or NSCLC-only models also requires further investigation. Nevertheless, by combining omics analysis and *in vivo* studies, this study has provided a comprehensive explanation of the molecular mechanism of MI-NSCLC comorbidity. Future studies should further define the mechanistic roles of these genes within comorbidity progression and assessing the feasibility of targeting them simultaneously for therapeutic intervention. Ultimately, this type of research may lead to the identification of early diagnostic biomarkers and improve clinical outcomes for patients suffering from this complex comorbid condition.

## 5 Conclusion

This study combined bioinformatics analysis with experimental validation to elucidate the critical functions of STEAP4, HP, TREM1, and CCR2 in the comorbidity of MI and NSCLC. The gene-based diagnostic model showed strong predictive capacity, indicating a potential method for the early diagnosis of comorbidity states. The findings emphasize the role of immune microenvironmental dysregulation in MI-NSCLC comorbidity. The genes that were identified were very closely linked with immune cell infiltration, particularly neutrophils and CD8^+^ T cells. The *in vivo* experiments again proved that STEAP4 plays a crucial role in comorbidity progression. Its upregulation led to the production of pro-inflammatory cytokines and imbalance in the immune system, which synergistically worsened cardiac dysfunction and tumor growth. Importantly, STEAP4 inhibition significantly improved cardiac function and suppressed tumor growth, indicating its potential as a target for therapy. Collectively, these results deepen current understanding of the molecular and immunological mechanisms underlying MI–NSCLC comorbidity and identify STEAP4, along with its associated gene network, as candidate biomarkers with diagnostic and therapeutic value. This work provides a conceptual framework for the development of targeted immunomodulatory strategies in complex cardiovascular–oncological comorbid conditions.

## Data Availability

The original contributions presented in the study are included in the article/supplementary material, further inquiries can be directed to the corresponding authors.
